# Trajectories of Handgun Carrying in Rural Communities From Early Adolescence to Young Adulthood

**DOI:** 10.1001/jamanetworkopen.2022.5127

**Published:** 2022-04-04

**Authors:** Alice M. Ellyson, Emma L. Gause, Sabrina Oesterle, Margaret R. Kuklinski, John S. Briney, Elizabeth H. Weybright, Kevin P. Haggerty, Vivian H. Lyons, Julia P. Schleimer, Ali Rowhani-Rahbar

**Affiliations:** 1 Department of Pediatrics, University of Washington, Seattle; 2 Firearm Injury and Policy Research Program, Harborview Injury Prevention and Research Center, University of Washington, Seattle; 3 Center for Child Health, Behavior, and Development, Seattle Children’s Research Institute, Seattle, Washington; 4 Southwest Interdisciplinary Research Center, School of Social Work, Arizona State University, Phoenix; 5 Social Development Research Group, School of Social Work, University of Washington, Seattle; 6 Department of Human Development, Washington State University, Pullman; 7 Department of Health Behavior and Health Education, University of Michigan, Ann Arbor; 8 Department of Epidemiology, University of Washington, Seattle

## Abstract

**Importance:**

Characterizing patterns of handgun carrying among adolescents and young adults can inform programs to reduce firearm-related harm. Longitudinal patterns of handgun carrying among rural adolescents have not been identified.

**Objectives:**

To assess specific points of intervention by characterizing patterns of handgun carrying by youths in rural communities from early adolescence to young adulthood and to quantify how age at initiation, duration, and frequency of carrying differ across identified patterns.

**Design, Setting, and Participants:**

This cohort study uses the control group of the community-randomized trial of the Communities That Care prevention system, conducted among public school students in 12 rural communities across 7 states. Participants self-reported their handgun carrying at 10 data collection points from 12 to 26 years of age (2005-2019). Data were analyzed from January to July 2021.

**Main Outcomes and Measures:**

Handgun carrying in the past 12 months. Latent class growth analysis was used to estimate handgun carrying trajectories.

**Results:**

In this longitudinal rural sample of 2002 students, 1040 (51.9%) were male; 532 (26.6%) were Hispanic, Latino, Latina, or Latinx; 1310 (65.4%) were White; and the highest level of educational attainment of either parent was a high school degree or less for 649 students (32.4%). The prevalence of handgun carrying in the last 12 months ranged from 5.3% (95 of 1795) to 7.4% (146 of 1969) in adolescence and increased during the mid-20s (range, 8.9% [154 of 1722] to 10.9% [185 of 1704] from 23 to 26 years of age). Among the participants who reported handgun carrying at least once between 12 and 26 years of age (n = 601 [30.0%]), 320 (53.2%) reported carrying a handgun in only 1 wave. Latent class growth analysis indicated 6 longitudinal trajectories: never or low probability of carrying (1590 [79.4%]), emerging adulthood carrying (166 [8.3%]), steadily increasing carrying (163 [8.1%]), adolescent carrying (53 [2.6%]), declining carrying (24 [1.2%]), and high probability and persistent carrying (6 [0.3%]). The earliest mean (SD) age at initiation of handgun carrying occurred in both the adolescent and declining carrying groups at the ages of 12.6 (0.9) and 12.5 (0.7) years, respectively. More than 20% of some groups (emerging adulthood [age 26 years: 49 of 154 (31.8%)], steadily increasing [age 26 years: 37 of 131 (28.2%)], declining [age 13 years: 7 of 23 (30.4%)], and high probability and persistent carrying [age 15 years: 3 of 6 (50.0%)]) reported carrying 40 times or more in the past year by the age of 26 years.

**Conclusions and Relevance:**

This study found distinct patterns of handgun carrying from adolescence to young adulthood in rural settings. Findings suggest that promoting handgun safety in rural areas should start early. Potential high-risk trajectories, including carrying at high frequencies, should be the focus of future work to explore the antecedents and consequences of handgun carrying in rural areas.

## Key Points

**Question:**

What are the patterns, frequency, and duration of handgun carrying among youths growing up in rural areas?

**Findings:**

This cohort study of 2002 rural students found 6 distinct trajectories of handgun carrying, with many youths initiating handgun carrying at least as early as 12 years of age, and more than 20% of some groups carrying a handgun 40 or more times in the past 12 months.

**Meaning:**

This study suggests that prevention programs to reduce the risk of firearm-related harm should be delivered early in the elementary school period.

## Teaser

This cohort study assesses specific points of intervention by characterizing patterns of handgun carrying by youths in rural communities from early adolescence to young adulthood and quantifies how age at initiation, duration, and frequency of carrying differ across identified patterns.

## Introduction

In 2019, suicide and homicide were the second and third leading causes, respectively, of death among individuals aged 12 to 26 years in US rural areas.^1^ Approximately 84% of homicides and 55% of suicides in this population involve a firearm.^2^ Firearm carrying, particularly handgun carrying, among adolescents is associated with multiple risk factors for interpersonal violence, including bullying, physical fighting, and assault, and increases the severity of injury and risk of death during episodes of interpersonal violence.^3,4,5,6^ Although not well studied, handgun carrying may also be associated with the risk of suicide or unintentional injury in this population. Addressing handgun carrying may help prevent fatal and nonfatal firearm-related harm.

Because handgun carrying is an important precursor of adolescent firearm-related harm,^7,8^ studying its longitudinal patterns can inform the timing of prevention programs. Longitudinal trajectories flexibly examine different behavioral patterns, the relative size of the groups, and may inform when, for whom, and on which risk factors to focus prevention efforts.^9^ Most studies of handgun carrying among adolescents and young adults have examined the behavior in urban settings,^9,10,11,12^ despite a high prevalence of handgun carrying among rural adolescents (3.6% in large metropolitan areas, 4.9% in small metropolitan areas, and 6.3% in nonmetropolitan areas).^13^ To our knowledge, there is little to no evidence on individual-specific variation patterns of handgun carrying duration or frequency in rural areas. Longitudinal patterns of handgun carrying among rural adolescents may inform whether existing urban prevention strategies apply in nonurban settings.

A scoping review found only 1 study explicitly examining handgun carrying among rural adolescents, and no studies characterized longitudinal patterns among rural adolescents.^14,15^ This is an important knowledge gap for several reasons.^4^ First, similar to urban adolescents, rural adolescents have a high level of exposure to firearms, but they also have high rates of formal training regarding firearm use and safety.^16,17,18^ Specific and effective strategies for preventing firearm-related harm among rural adolescents cannot be identified and implemented without a greater understanding of and respect for rural firearm culture and the motivations for specific forms of firearm use. Second, the characteristics and severity of firearm-related harm among adolescents differ in urban and rural settings. Rates of firearm suicide are higher in rural areas than in urban areas.^19^ Third, the health consequences of handgun carrying may differ for rural adolescents because health care is harder to access in rural communities. These differences are important to rural health care practitioners who rely largely on evidence from urban settings to inform prevention.

We sought to (1) characterize longitudinal patterns of handgun carrying from adolescence to young adulthood and (2) assess the age at initiation, duration, and frequency of handgun carrying among youths in rural areas. This study focuses on initiation, duration, and frequency of handgun carrying because prior research indicates that initiation of carrying is a key inflection point in involvement in violence as well as a target for primary prevention.^15^ These objectives will help us to demonstrate whether patterns of handgun carrying among youths in rural areas are qualitatively similar to those identified in urban areas.^9,10^ If handgun carrying trajectories are similar in rural and urban settings, age-specific interventions of handgun carrying may be effective in both settings. If distinct rural patterns of handgun carrying among youths emerge and differ from those observed among urban adolescents, it will bolster evidence for tailoring prevention to adolescents in rural communities.

## Methods

### Study Design, Participants, and Setting

The study sample comprised 2002 participants in the control group of the Community Youth Development Study (CYDS), a community-randomized trial of the Communities That Care prevention system in 12 communities across 7 states (Colorado, Illinois, Kansas, Maine, Oregon, Utah, and Washington).^20,21,22^ All communities were rural incorporated towns (total populations in 2003 ranged from 1500 to 41 000). The CYDS is a longitudinal, grade cohort study of fifth-grade public school students in the 2003-2004 school year. Written parental consent and written student assent were obtained for 76.4% of the eligible population (2002 of 2621).^4^ Race and ethnicity were self-reported by participants during adolescence to understand the sociodemographic characteristics of the participants and communities participating in the study. Participants were asked “Are you Spanish/Hispanic/Latino?” with the option to select yes or no and were asked “What is your race?” with the option to select all that apply from the following list: White or Caucasian, Black or African American, Asian, American Indian or Alaska Native, Native Hawaiian or Pacific Islander, or other (please specify). The CYDS repeatedly collected survey data from this grade cohort, with 89.8% retention (1797 of 2001 active, living participants) through grade 12 (2011; mean [SD] cohort age, 18.1 [0.4] years) and 86.4% retention (1711 of 1980 active, living participants) through the age of 26 years (2019). The University of Washington Human Subjects Review Committee approved this protocol. This study follows the Strengthening the Reporting of Observational Studies in Epidemiology (STROBE) reporting guideline for cohort studies.

### Measures

#### Handgun Carrying

Participants self-reported their handgun carrying at 10 data collection points from 12 to 26 years of age (2005-2019). From 12 to 19 years of age, respondents were asked, “How many times in the past year (12 months) have you carried a handgun?” Response options were the following ordinal categories: never, 1 to 2 times, 3 to 5 times, 6 to 9 times, 10 to 19 times, 20 to 29 times, 30 to 39 times, or 40 or more times. From 21 to 26 years of age, the question stem was revised to ask, “How many times in the past year (12 months) have you carried a handgun other than while hunting or as part of your job?” The ordinal response options did not change. For analyses, ordinal responses were dichotomized so that 0 indicated never carrying a handgun in the past year and 1 indicated carrying a handgun at least once in the past year.

#### Age at Initiation and Duration

The age at initiation of handgun carrying was calculated using the interview age in the first study wave that each participant reported handgun carrying. Duration of handgun carrying was calculated as the number of study waves that each participant reported handgun carrying even if the carrying was not in consecutive waves.

### Statistical Analysis

Data were analyzed from January to July 2021. Latent class growth analysis was used to estimate handgun carrying trajectories using Mplus software, version 8 (Muthen & Muthen).^23^ These models were implemented using the full information maximum likelihood estimation, making use of all available data and robustly modeling trajectories with missing responses across waves. All 2002 study participants were included in the analyses. Data on handgun carrying were collected from the age of 12 years (grade 6) to the age of 26 years. In latent class growth analysis analyses, the unit of time was the mean age for the cohort at each data collection point, ranging from 12 years of age in 2005 to 26 years of age in 2019. Handgun carrying responses were available at all data collection points for 59.8% of the sample (n = 1197). Of those with missing data (n = 805), nearly half (398 [49.4%]) had missing data at 1 wave, 272 (33.8%) had missing data at 2 to 3 waves, and 135 (16.8%) had missing data at 4 or more waves. First, the best-fitting and most parsimonious linear model was identified based on bayesian information criterion (BIC) and based on results from the Vuong-Lo-Mendell-Rubin test and the bootstrapped likelihood ratio test.^23,24^ Next, the BIC from the identified linear model was compared with the BIC from a higher-ordered polynomial model (quadratic), and the model with the minimum BIC was selected. We used 1000 random starting values. See eAppendix 1 and eAppendix 2 in the Supplement for additional information about model specification, selection, and evaluation, which follow 2017 guidelines for reporting on latent trajectory studies (GRoLTS).^24^ Participants were then assigned to trajectory groups based on the highest posterior probability of group membership. Then, the age at initiation, duration, and frequency of handgun carrying for each group were summarized and compared descriptively. These trajectories were detected in our study sample. Participants did not actually belong to a particular group, the groups may have changed over time or may be different in other populations, and each of these groups has been estimated with uncertainty (eTable 2 in the Supplement).^25,26^

### Sensitivity Analysis

Prior work in urban areas has estimated longitudinal trajectories only among those who reported handgun carrying.^9^ To compare identified trajectories in this study with these prior findings, an alternate specification excluding participants who never reported handgun carrying was also estimated. We assessed whether rural handgun carrying patterns varied when participants who never reported handgun carrying were excluded.

## Results

**Figure 1.**
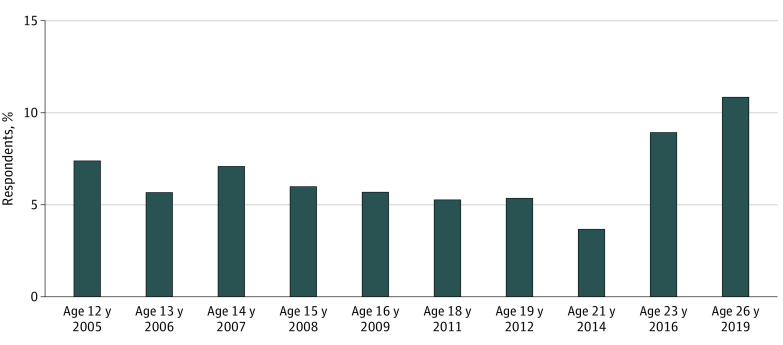
Percentage of Respondents Reporting Handgun Carrying at Least Once in the Past 12 Months at Each Age Beginning at the age of 21 years, respondents were prompted to exclude handgun carrying while hunting or as part of their job.

Study participants were a mean age of 12 years in grade 6 in 2005. A total of 962 students (48.1%) were female, and 1040 (51.9%) were male; 532 (26.6%) were Hispanic, Latino, Latina, or Latinx; 1310 (65.4%) were White; and the highest level of educational attainment of either parent was a high school degree or less for 649 youth participants (32.4%) (Table). A total of 1401 participants (70.0%) reported never carrying a handgun, while 601 participants (30.0%) reported carrying a handgun at least once between 12 and 26 years of age. Among those who reported carrying in at least 1 study wave, 320 (53.2%) reported carrying in only 1 wave. The prevalence of handgun carrying in the past 12 months ranged from 3.7% (66 of 1788) to 7.4% (146 of 1969) of the study sample between 12 and 21 years of age and increased in young adulthood (Figure 1).

**Table.** Study Sample Demographic Information by Carrying Status

Characteristic	Adolescents and young adults reported carrying, No. (%)	
	Never (n = 1401 [70.0%])	At least once (n = 601 [30.0%])
Gender		
Male	589 (42.0)	451 (75.0)
Female	812 (58.0)	150 (25.0)
Highest level of education of any parent		
Grade school or less	57 (4.1)	21 (3.5)
Some high school	119 (8.5)	43 (7.2)
Completed high school	282 (20.1)	127 (21.1)
Some college	316 (22.6)	150 (25.0)
Completed college	398 (28.4)	157 (26.1)
Graduate or professional degree	159 (11.4)	80 (13.3)
Missing	70 (5.0)	23 (3.8)
Hispanic ethnicity		
Yes	373 (26.6)	159 (26.5)
No	1028 (73.4)	442 (73.5)
Race^a^		
American Indian or Alaska Native	77 (5.5)	39 (6.5)
Asian	26 (1.9)	17 (2.8)
Black or African American	50 (3.8)	17 (2.8)
Native Hawaiian or Pacific Islander	10 (0.7)	5 (0.8)
White or Caucasian	922 (65.8)	388 (64.6)
Other	379 (27.1)	167 (27.8)

^a^ Race categories were not mutually exclusive, and data on race were missing for 19 participants. There were 357 participants who selected “Other” for race but did not provide a write-in response. Among these participants, 277 indicated “Yes” in the separate question asking “Are you Spanish/Hispanic/Latino?” with response options yes or no. There were 189 participants who selected “Other” for race and provided a write-in response. Among these participants, 19 provided a response that included the word “Spanish,” 58 provided a response that included the word “Hispanic,” and 17 provided a response that included the word “Latin.” Some also provided responses with nationalities.

### Rural Handgun Carrying Trajectories

**Figure 2.**
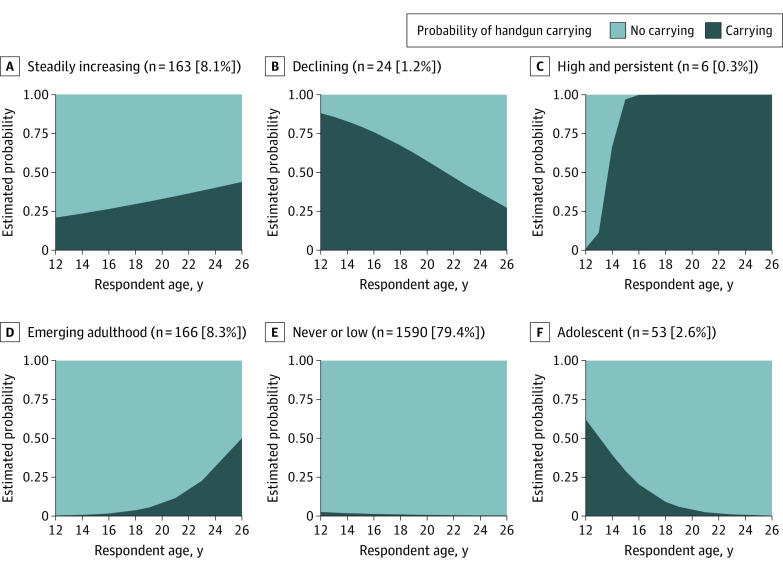
Estimated Probability of Handgun Carrying at Each Age by Latent Trajectory Group Membership

Using latent class growth analysis, we identified a linear model with 6 handgun carrying trajectories (Figure 2) as the best-fitting and most parsimonious model using criteria in eFigure 1, eTable 1, and eTable 2 in the Supplement. The most common pattern, never or low probability carrying (1590 [79.4%]), consisted of very low probabilities of handgun carrying between 12 and 26 years of age. It included those who reported never carrying from 12 to 26 years of age and also included 189 participants who reported handgun carrying in only 1 wave. The emerging adulthood carrying trajectory (166 [8.3%]) had low estimated probabilities of carrying in adolescence (<0.05 before 18 years of age) but increased quickly thereafter to 0.50 (95% CI, 0.20-0.80) at 26 years of age. A pattern of steadily increasing carrying (163 [8.1%]) was defined by steadily climbing probabilities of handgun carrying from 0.21 (95% CI, 0.13-0.29) at 12 years of age to 0.44 (95% CI, 0.34-0.53) at 26 years of age. Adolescent carrying (53 [2.6%]) was distinguished by a high estimated probability (0.62 [95% CI, 0.33-0.92]) of handgun carrying at 12 years of age, but quickly decreased to almost zero by 18 years of age. This pattern was different from declining carrying (24 [1.2%]), where the mean estimated probability of carrying was also quite high at 12 years of age (0.88 [95% CI, 0.77-0.99]) but remained likely in early adulthood at 21 years of age, with a mean estimated probability of 0.52 (95% CI, 0.27-0.77). Last, the mean estimated probability of carrying for the high and persistent carrying pattern (6 [0.3%]) increased rapidly from 0.01 (95% CI, −0.04 to 0.05) at 12 years of age to 0.998 (95% CI, 0.99-1.00) at 16 years of age and remained almost certain throughout young adulthood.

### Initiation, Duration, and Frequency of Handgun Carrying by Trajectory Group Membership

The earliest mean (SD) age at initiation occurred in the adolescent and declining carrying groups at the ages of 12.6 (0.9) and 12.5 (0.7) years, respectively. Approximately 24.3% of youths who ever reported carrying (146 of 601) carried a handgun in the 12 months prior to grade 6, at or before the age of 12 years, suggesting the age at initiation of handgun carrying in rural areas is young. Three of the trajectories (steadily increasing, high and persistent, and never or low) had a mean (SD) age at initiation of 14.6 (2.3) years, 14.6 (1.2) years, and 14.6 (2.3) years, respectively and the mean (SD) age at initiation in the emerging adulthood carrying group was 23.9 (2.2) years.

**Figure 3.**
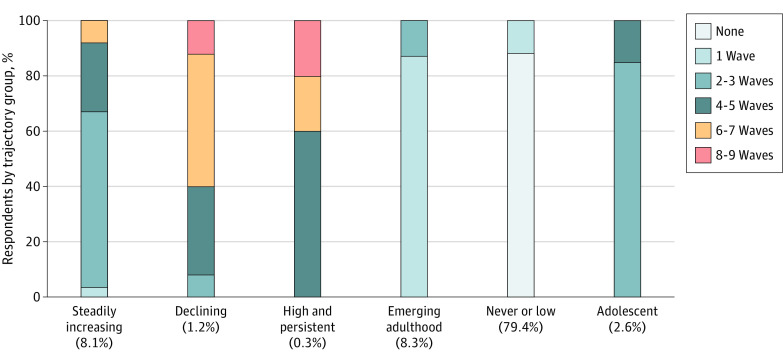
Duration of Handgun Carrying: Percentage of Respondents Reporting Handgun Carrying in Multiple Study Waves by Trajectory Group

Those most likely to be in the high and persistent group reported handgun carrying in more than half the study waves (Figure 3). Similarly, 95.8% of declining carriers (23 of 24) reported carrying in 4 or more waves. Those most likely to be in other groups (never or low, emerging adulthood, adolescent, and steadily increasing) reported carrying in fewer waves.

**Figure 4.**
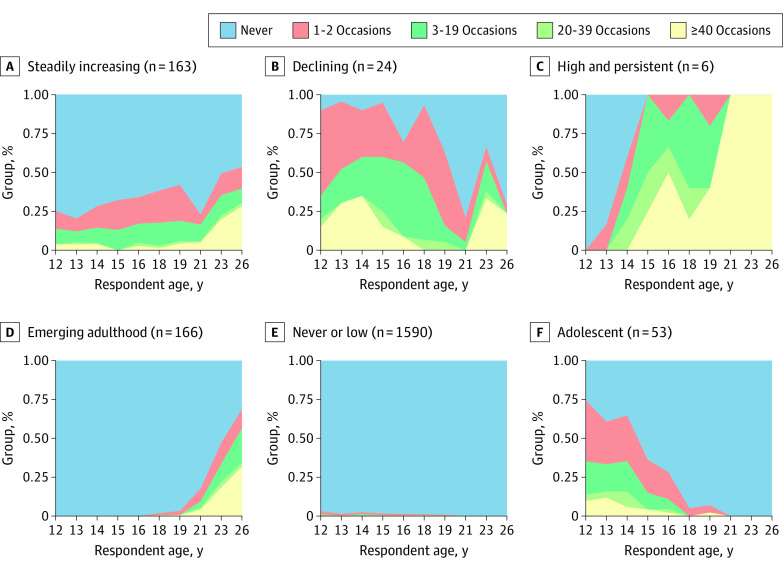
Frequency of Handgun Carrying by Trajectory Group and Age

Handgun carrying occurred with varying past-year frequency across and within trajectory groups (Figure 4); 11.8% (6 of 51) of those most likely to follow the adolescent carrying pattern reported handgun carrying 40 or more times at 13 years of age, but the percentage carrying at this frequency decreased to 2.2% (1 of 46) by 16 years of age. A considerable portion of carrying among those in the steadily increasing, declining, and adolescent trajectories was infrequent, at approximately 1 to 2 occasions in the last 12 months. Generally, the participants most likely to be on trajectories with greater carrying probabilities (high and persistent and declining) tended to carry more frequently in a year than those in other trajectory groups. More than 20% of some groups (emerging adulthood [age 26 years: 49 of 154 (31.8%)], steadily increasing [age 26 years: 37 of 131 (28.2%)], declining [age 13 years: 7 of 23 (30.4%)], and high probability and persistent carrying [age 15 years: 3 of 6 (50.0%)]) reported carrying 40 times or more in the past year by the age of 26 years.

### Sensitivity Analysis

Identified trajectories were similar to primary analysis results when participants who never carried were excluded (eFigure 2 in the Supplement). Steadily increasing, declining, high and persistent, emerging adulthood, and adolescent carrying trajectories were still distinctly identified. The never or low probability trajectory was not identified. The 189 participants who were assigned to the never or low probability trajectory in the primary analysis were reassigned to 1 of the other trajectories in this sensitivity analysis, so the size of these groups changed somewhat (eAppendix 3 in the Supplement).

## Discussion

We found 6 distinct patterns of handgun carrying from adolescence to young adulthood in this rural sample. The most common trajectory had a pattern of never or low probability of handgun carrying. Three trajectories indicated an increasing likelihood of handgun carrying, and the remaining 3 trajectories showed low carrying probabilities. The mean age at initiation of handgun carrying varied, with the earliest at approximatley 12 years of age (adolescent and declining trajectories), and the latest at 23 years of age (emerging adulthood trajectory). The frequency and duration of handgun carrying also differed by trajectory, with declining and high and persistent patterns defined by handgun carrying for longer and more often than the other trajectories. These patterns depicting when and how often adolescents growing up in rural areas carry handguns are salient because averting the behavior is key for prevention.^15^ Future work should examine strategies that delay initiation, shorten duration, and reduce frequency of handgun carrying.

The prevalence of handgun carrying during young adulthood was greater in this rural sample than in urban areas.^27,28,29^ Handgun carrying increased among young adults who grew up in rural areas to 8.9% at 23 years of age and 10.9% at 26 years of age, while it remained between 4% and 6% among urban young adults through 30 years of age. The literature on urban areas finds 4 distinct handgun carrying trajectories from 15 to 29 years of age—declining, bell-shaped, late-initiating, and persistent trajectories.^9,10^ Some of those trajectories are similar to those found in the present study of rural communities, including adolescent carrying (declining trajectory in urban literature), emerging adulthood carrying (late-initiating trajectory in urban literature), and high and persistent carrying, but our study also identified a steadily increasing trajectory.

The differences between our findings and those for urban handgun carriers point to the need for prevention approaches tailored for rural contexts. We identified a steadily increasing handgun carrying trajectory in rural areas, with no peak through 26 years of age, compared with the bell-shaped trajectory group peaking at 21 years of age among urban carriers.^9,10^ In rural sensitivity analyses, steadily increasing carrying comprised more than half of participants who ever reported carrying (51.1% [307 of 601]) compared with approximately one-third (35.6% [561 of 1574]) of the sample in urban communities following a bell-shaped handgun carrying trajectory. Although the same intervention timing to address initiation may be suitable in both settings (approximately 12-14 years of age), the circumstances leading to a bell-shaped trajectory of handgun carrying compared with a steadily increasing trajectory of handgun carrying may be different and should be considered in future work. Similar deviations between urban and rural areas were present in the high and persistent trajectories. In the high and persistent handgun carrying group in rural areas, carrying probabilities and frequencies were much greater, with a probability close to 1.0 and many youths carrying 40 times or more within the past year, through late adolescence and young adulthood, compared with lower estimated probabilities in urban areas (probability, 0.3 at 15 years of age and 0.6 at 21 years of age). Although this high and persistent handgun carrying group was small and less than 1.0% of carriers in rural settings, the high frequency and duration of carrying may indicate high risk and may present a key point of intervention. Future research should examine whether carrying trajectories are associated with firearm-related harm and the antecedents and consequences of handgun carrying in rural contexts.

Understanding trajectories can be useful in informing and tailoring firearm carrying prevention programs. Given the early age of handgun carrying initiation in rural areas for most trajectories,^30^ programs established for educational and youth-serving organizational settings to educate adolescents about firearms, firearm violence, and how to resolve conflicts without firearms may be suitable for rural areas, especially if those programs connect to the firearm culture of that community. Almost all existing interventions that seek to reduce general weapon carrying focus on factors associated with crime.^15^ This framework may not apply to all or even most youths in rural settings, and these programs may not be appropriate for youths engaging in handgun carrying with different motivations or circumstances. Other studies support the hypothesis that cultural norms around firearm use may play a large role in addressing adolescents’ firearm safety.^17,18^ Qualitative studies exploring rural youths’ motivations and circumstances of carrying and how to design prevention programs for a rural context are needed. Firearm-specific programs, such as Straight Talk About Risks and Hands Without Guns, could be adapted for rural contexts and implemented to achieve these goals.^31,32^ The Communities That Care prevention system, which assists communities in identifying and addressing local risks to positive adolescent development, could also be harnessed to address firearm safety challenges.^20,21^

### Limitations

This study has some limitations. The survey asked about carrying of handguns only and not all firearms. Because long-gun carrying may be more common in rural areas, this is an important distinction warranting further study. Handgun carrying responses were obtained by self-report and may be subject to recall and social desirability biases. This limitation was mitigated by study protocols that removed suspect survey responses (eg, failing attention or honesty checks). Participants were not directly asked for the age at which they started carrying, so it was not possible to identify youth or childhood experiences earlier than 12 years of age. This left censoring of the data on the initiation of handgun carrying is a limitation of both this study and existing firearm carrying studies. Future work should seek to identify firearm involvement prior to adolescence in rural areas. The multistate study was not a representative sample, so results may not be generalizable to all rural areas in the US. Some study participants moved away from rural communities either during adolescence or in young adulthood, so results describe handgun carrying trajectories among young people who grew up in rural areas. Last, the wording of the handgun carrying question changed starting at 21 years of age to exclude some contexts (ie, handgun carrying other than while hunting or as part of a job). This change likely led to a reduced prevalence of handgun carrying reported by the sample during young adulthood relative to adolescence and may have influenced the trajectories identified if the circumstances of carrying varied across trajectory groups.

We could not distinguish between legal and illegal handgun carrying. Five of the 7 states (Colorado, Maine, Oregon, Utah, and Washington) had a right-to-carry law in effect throughout the duration of the study period.^33^ The remaining 2 states (Kansas and Illinois) implemented right-to-carry laws in 2007 and 2014, respectively. Although handgun possession at ages younger than 18 years is barred by US federal law, circumstances are exempted (eg, in the course of employment, such as ranching or farming, with prior written consent of a parent) that were not measured in this study. Because the survey did not contain information on why participants carried, we did not measure the extent of potential bias from changing the wording of questions or other factors.

## Conclusions

To our knowledge, this is one of the first studies to identify longitudinal patterns of handgun carrying from adolescence to young adulthood in rural areas. For the 30% of participants who carried a handgun, the age at initiation was young, starting at 12 to 14 years. Prevention programs to reduce the risk of firearm-related harm in this population may need to start early, in the elementary school period. We identified a few carrying patterns with high or above-average carrying probabilities. These patterns may be associated with greater risk of firearm-related harm owing to more frequent and longer duration of carrying and may be suitable for focused prevention programs.

## Notes

Supplement. **eAppendix 1.** Model Specification and Selection **eFigure 1.** Information Criterion Values by the Number of Latent Classes **eTable 1.** Statistical Selection Criteria for the Number of Trajectory Groups **eAppendix 2.** Model Evaluation **eTable 2.** Average Posterior Probability of Assignment by Trajectory Group Membership **eAppendix 3.** Sensitivity Analysis **eFigure 2.** Estimated Probability of Handgun Carrying at Each Age by Latent Trajectory Group Membership Only Among Those Who Ever Report Handgun Carrying
eReferences.
