# Supplementary material for: Trajectories of Handgun Carrying in Rural Communities From Early Adolescence to Young Adulthood
Source: JAMA Netw Open. 2022 Apr 4;5(4):e225127. doi: 10.1001/jamanetworkopen.2022.5127 (PMC8980900; doi:10.1001/jamanetworkopen.2022.5127)
Supplement: Supplement. — eAppendix 1. Model Specification and Selection eFigure 1. Information Criterion Values by the Number of Latent Classes eTable 1. Statistical Selection Criteria for the Number of Trajectory Groups eAppendix 2. Model Evaluation eTable 2. Average Posterior Probability of Assignment by Trajectory Group Membership eAppendix 3. Sensitivity Analysis eFigure 2. Estimated Probability of Handgun Carrying at Each Age by Latent Trajectory Group Membership Only Among Those Who Ever Report Handgun Carrying eReferences. [file jamanetwopen-e225127-s001.pdf]

Supplementary Online Content

Ellyson AM, Gause EL, Oesterle S, et al. Trajectories of handgun carrying in rural communities from early adolescence to young adulthood. *JAMA Netw Open.* 2022;5(4):e225127. doi:10.1001/jamanetworkopen.2022.5127

eAppendix 1. Model Specification and Selection
eFigure 1. Information Criterion Values by the Number of Latent Classes
eTable 1. Statistical Selection Criteria for the Number of Trajectory Groups
eAppendix 2. Model Evaluation
eTable 2. Average Posterior Probability of Assignment by Trajectory Group Membership
eAppendix 3. Sensitivity Analysis
eFigure 2. Estimated Probability of Handgun Carrying at Each Age by Latent Trajectory Group Membership Only Among Those Who Ever Report Handgun Carrying
eReferences.

This supplementary material has been provided by the authors to give readers additional information about their work.

© 2022 Ellyson AM et al. *JAMA Network Open.*

eAppendix 1. Model Specification and Selection

Model specification. Our analyses include all 2,002 participants. Prior research has applied inclusion criteria that requires participants to report handgun carrying in at least one wave to be included in analyses and thereby considered handgun carrying trajectories only among those who ever report carrying.[1,2] This approach prioritizes the identification of timing markers to aid public health prevention that is implemented at a particular point in adolescence or young adulthood. However, there are several advantages of assessing trajectories among ***all*** youth. First, including all youth avoids conditional inclusion criteria. Setting inclusion criteria based on behavior at any time in the study period makes inclusion in the study conditional on a behavior that is not known at baseline, before the onset of follow-up waves. All results are conditional on this inclusion criteria. Including all youth in the sample allows all described probabilities to be unconditional. Second, this approach handles intermittent missingness well. If the sample is restricted to only youth who ever report carrying, some youth may be mislabeled as a "never carrier" since there are a large proportion, 40.2%, for whom at least one wave of handgun carrying information is missing. Youth can only be accurately labeled a "never carrier" if their response to carrying is observed in all study waves. Lastly, empirically estimating whether a group of low or never carriers is its own group is an important part of informing prevention. It provides a clear, natural, and empirically validated comparison to understand both the antecedents and the consequences of handgun carrying. This approach prioritizes identifying risk and protective areas where an appropriate comparison group can be used to understand differences between a low risk group and higher risk groups. Handgun carrying trajectory subgroups are not identified ex ante by other measured demographic or socioeconomic characteristics or other covariates. This choice is intentional and data-driven, and evaluates trajectories present in the sample using only observed handgun carrying patterns. This approach is designed to identify specific points and subgroups of intervention based on three critical components of handgun carrying patterns, namely initiation age of carrying, frequency of carrying, and duration of carrying behavior.

© 2022 Ellyson AM et al. *JAMA Network Open.*

LCGA is the modeling approach used in all specifications. LCGA is a longitudinal growth model that uses class-specific fixed effects to capture discrete differences over time across groups.[3,4] LCGA takes advantage of sequential patterns of time and permits variance across time and across trajectory groups compared to traditional latent class analysis with repeated measures. Individual-specific deviations from the latent class mean are treated as residual error.[3] Standard errors in all specifications were clustered at the participant level to account for serial correlation in handgun carrying. LGMM (latent growth mixture modelling), which permits variance within classes, was considered. If handgun carrying was a continuous variable with an open numeric response option, LGMM would take advantage of slight variations in reported handgun carrying. However, with both ordinal and binary measures from the CYDS survey data, these variations already binned in the data. Traditional latent class analysis was also considered, but LCGA takes advantage of the sequential pattern of time which is critical to understanding longitudinal handgun carrying.

Model selection. Both statistical criteria and prevention considerations were used to determine the model that best characterized the number of trajectory groups. Statistical selection criteria for trajectory models include the Akaike information criterion (AIC), Bayesian information criterion (BIC), and sample size adjusted Bayesian information criterion (aBIC) with the goal of minimizing information loss. Lower AIC, BIC, and aBIC values tend to indicate models with less information loss that are a better fit to the data. There is a consensus among experts that BIC is favored in latent trajectory analyses for selecting the best fitting number of classes.[4] A 1-class model was first fit and then additional classes were modeled until the lowest BIC value was identified to determine the best number of classes. To determine whether there was adequate separation between classes in models with k compared to k-1 classes, we conducted the Vuong-Lo-Mendell-Rubin test (VLMR) as well as the bootstrap likelihood ratio test (BLRT) on the model with the best number of classes. Research also suggests that applied researchers tend to reduce the number of latent trajectories to a lower number that would still be theoretically important or meaningful in prevention practice when the optimal number of classes

© 2022 Ellyson AM et al. *JAMA Network Open.*

identified by model selection tools and the entropy index are large, when these tools conflict with each other, or when they conflict with theory.[4] When the number of participants in each latent class becomes small, the ability to meaningfully design prevention and intervention programs becomes more difficult. Statistical criteria were prioritized, and prevention criteria were considered.

AIC, BIC, and sample size adjusted BIC suggest the 6-class model is the best fit (**eFigure 1 and eTable 1**). While the AIC continues to decline with the addition of a 7th class, the relative difference is quite small and conflicts with minimum values for both BIC and aBIC at 6 classes. Both the Vuong-Lo-Mendell-Rubin test (VLMR) and Bootstrapped likelihood ration test (BLRT) also support the 6-class model (**eTable 1**) and reject the null hypothesis that the 5-class model is a better fit than the 6-class model. While the six-class model and the last trajectory group of *high and persistent* carriers is small, n = 6 participants (1.0% of CYDS control community youth who ever reported carrying and 0.3% of participants overall), this group has different estimated carrying probabilities, carrying frequencies, and carrying durations which may be important in prevention practice. Since this group seems meaningful in prevention practice and the statistical criteria all support the 6-class model, the 6-class model was chosen.

© 2022 Ellyson AM et al. *JAMA Network Open.*

eFigure 1. Information Criterion Values by the Number of Latent Classes

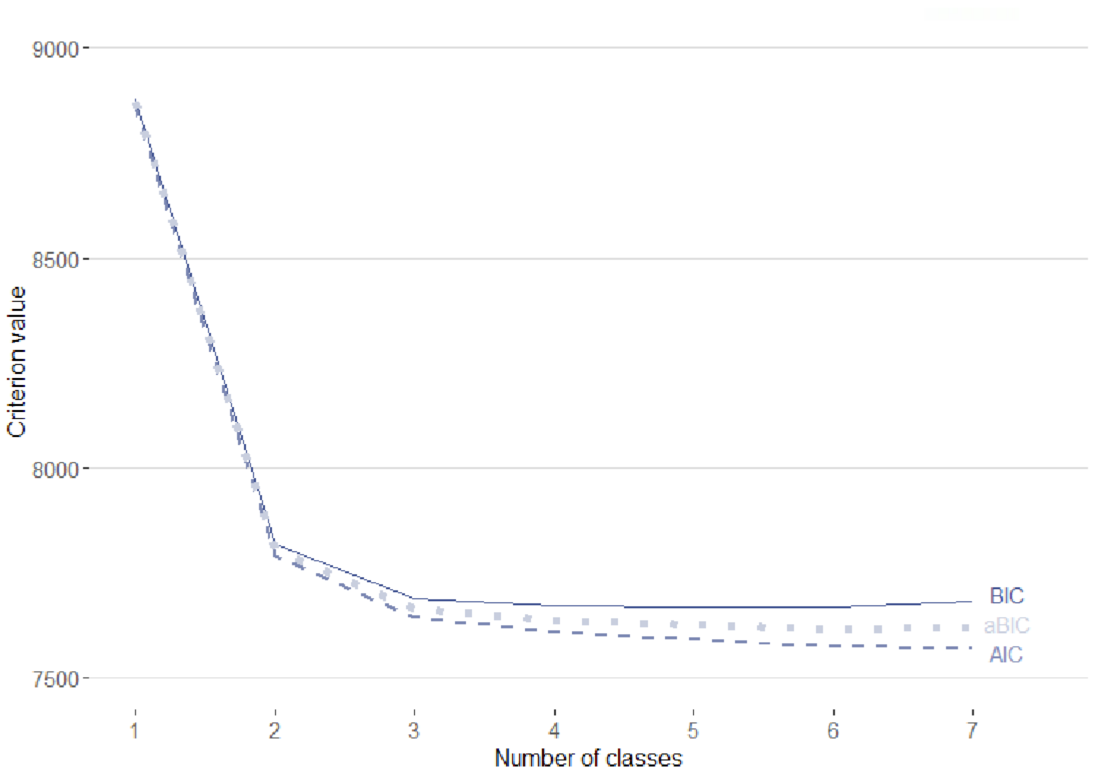

© 2022 Ellyson AM et al. *JAMA Network Open.*

eTable 1. Statistical Selection Criteria for the Number of Trajectory Groups

Number of classes	BIC	aBIC	AIC	VLMR p-value	BLRT p-value	Entropy
1	8877.352	8870.997	8866.148			--
2	7820.512	7804.627	7792.503			0.846
3	7690.046	7664.629	7645.230			0.714
4	7672.233	7637.285	7610.612			0.644
5	7670.552	7626.073	7592.125			0.757
**6**	**7670.233**	**7616.223**	**7575.001**	0.0205	0.0000	0.782
7	7683.351	7619.809	7571.313			0.724

© 2022 Ellyson AM et al. *JAMA Network Open.*

eAppendix 2. Model Evaluation

Model evaluation. Two criteria were used in model evaluation, entropy and average posterior probabilities of assignment. Entropy is a measure of classification uncertainty in class assignment where higher values represent a better fit to the modeled profiles and less classification uncertainty.[4] While entropy is informative about changes in the amount of classification uncertainty that may be introduced in one model compared to another, but models with higher entropy are only favored when selecting among models with similar relative fit indices.[4,5] Values closer to 1 provide supporting evidence that profile classification of individual participants occurs with minimal uncertainty.[6] Entropy values suggest that there is some classification uncertainty in the selected model. Entropy is highest with the 2-class solution. Among solutions with the best BICs, entropy is higher from the 6-class solution suggesting that there is less classification uncertainty in the 6-class solution compared to other class solutions with similar BICs.[5]

In addition, the Average Posterior Probability of Assignment (APPA) is the average posterior probability of belonging to class k over all the individuals assigned to class k and is often described as the average latent class probabilities for the most likely latent class membership.[4] Values closer to 1 are preferred and ideally greater than 0.7. Average Posterior Probabilities of Assignment (APPA) meet the suggested threshold of 0.7 (**eTable 2**). The highest is 0.905 (Class 5), the average APPA for all classes is 0.8135, and the lowest APPA is 0.693 (Class 6).

© 2022 Ellyson AM et al. *JAMA Network Open.*

eTable 2. Average Posterior Probability of Assignment by Trajectory Group Membership

APPA	1	2	3	4	5	6
**1** *(steadily increasing)*	**0.809**	0.028	0.003	0.082	0.033	0.046
**2** *(declining)*	0.116	**0.828**	0.017	0	0	0.039
**3** *(high and persistent)*	0.097	0.032	**0.871**	0	0	0
**4** *(emerging adulthood)*	0.135	0	0	**0.775**	0.089	0.001
**5** *(never or low)*	0.019	0	0	0.062	**0.905**	0.014
**6** *(adolescent)*	0.181	0.051	0	0	0.074	**0.693**

Note. Row provides the assigned class, columns provide the APPA.

© 2022 Ellyson AM et al. *JAMA Network Open.*

eAppendix 3. Sensitivity Analysis

The sensitivity analysis which imposed conditional inclusion criteria for those who ever reported carrying found results that were similar to the primary analysis among all sampled youth.Statistical selection criteria, minimizing BIC, identified the 5-class solution as the best number of classes which identified five trajectory groups very similar to those from the main analysis without inclusion criteria - the *never or low* carrying group from the primary analysis was omitted by construction. The sizes of the five trajectory groups changed because of the reassignment of 189 participants in the *never or low* group in the primary analysis. The group of *steadily increasing carriers* grew from 163 participants to 307 participants and comprised over half (51.1%) of all carriers. The *adolescent carrier* group also increased from 53 participants to 156 participants or 26.0% of all carriers. The group of declining carriers also increased slightly, from 24 to 39 participants, and represents 6.5% of carriers in the sample. The *high and persistent carrying* group remained the same, with the same 6 participants identified, and the *emerging adulthood* group declined in size from 166 participants to only 93 participants, about 15.5% of all carriers. In addition, the estimated probabilities for the *emerging adulthood carrying* group in this specification were quite different, remaining at almost zero from age 12 to age 21 and then increasing very rapidly to almost 1.0 by age 26, suggesting that the changes may be driven by a smaller number of late-initiating consistent carriers in early adulthood being assigned to this group.

© 2022 Ellyson AM et al. *JAMA Network Open.*

eFigure 2. Estimated Probability of Handgun Carrying at Each Age by Latent Trajectory Group Membership Only Among Those Who Ever Report Handgun Carrying

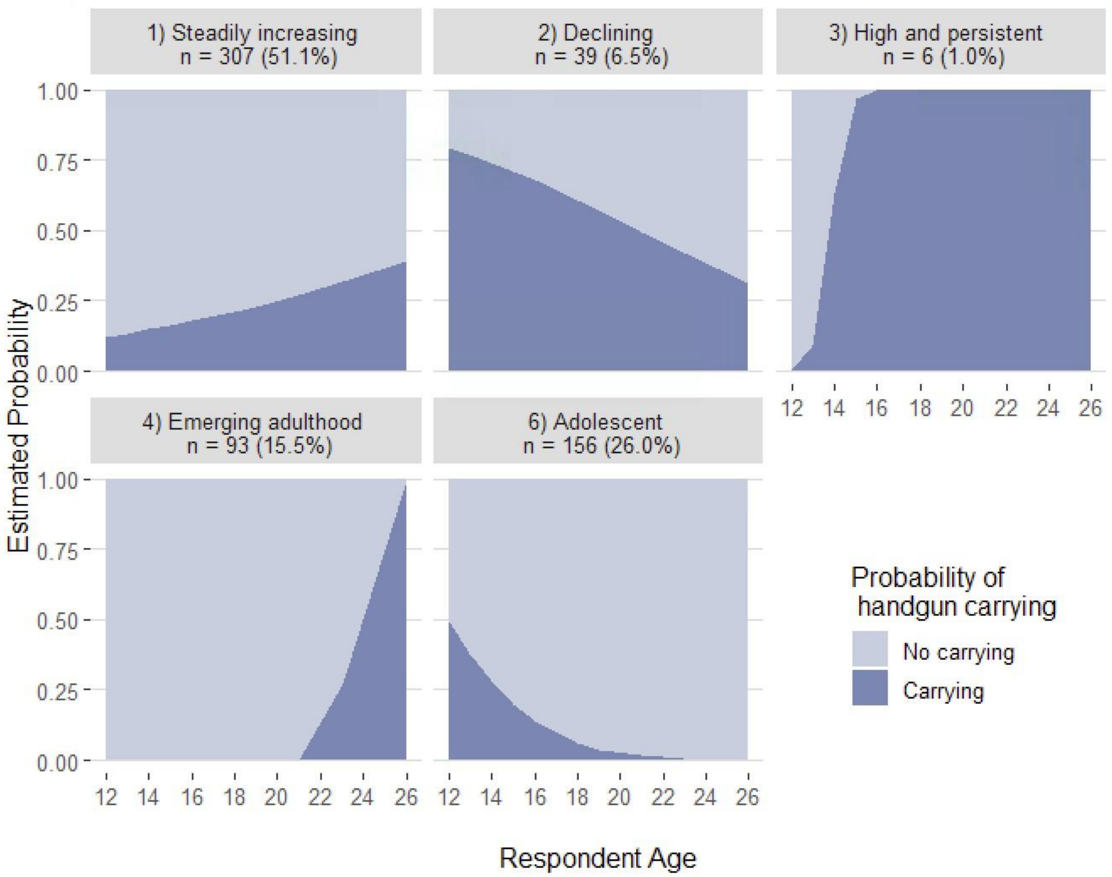

Note. Only 601 participants who ever report carrying included.

eReferences.

1. Dong B, Jacoby SF, Morrison CN, Wiebe DJ. Longitudinal heterogeneity in handgun-carrying behavior among urban American youth: intervention priorities at different life stages. J Adolesc Health. 2019;64(4):502-508. Medline:30455034 doi:10.1016/j.jadohealth.2018.09.026

2. Dong B. Developmental comorbidity of substance use and handgun carrying among U.S. youth. Am J Prev Med. 2021;61(2):209-216. Medline:33958238 doi:10.1016/j.amepre.2021.02.015

3. van der Nest G, Lima Passos V, Candel MJJM, van Breukelen GJP. An overview of mixture modelling for latent evolutions in longitudinal data: modelling approaches, fit statistics and software. Adv Life Course Res. 2020;43:100323. doi:10.1016/j.alcr.2019.100323

4. van de Schoot R, Sijbrandij M, Winter SD, Depaoli S, Vermunt JK. The GRoLTS-checklist: guidelines for reporting on latent trajectory studies. Struct Equ Modeling. 2017;24(3):451-467. doi:10.1080/10705511.2016.1247646

5. Ram N, Grimm KJ. Growth mixture modeling: a method for identifying differences in longitudinal change among unobserved groups. Int J Behav Dev. 2009;33(6):565-576. Medline:23885133 doi:10.1177/0165025409343765

6. Celeux G, Soromenho G. An entropy criterion for assessing the number of clusters in a mixture model. J Classif. 1996;13:195-212. doi:10.1007/BF01246098

© 2022 Ellyson AM et al. *JAMA Network Open.*
